# Patient reported outcome measures and cardiovascular outcomes following high dose modern intravenous iron in non-dialysis dependent chronic kidney disease: secondary analysis of ExplorIRON-CKD

**DOI:** 10.1038/s41598-023-44578-6

**Published:** 2023-10-26

**Authors:** Xenophon Kassianides, Sunil Bhandari

**Affiliations:** Academic Renal Research Department, Hull University Teaching Hospitals NHS Trust and the Hull York Medical School, Kingston upon Hull, UK

**Keywords:** Medical research, Nephrology

## Abstract

Intravenous iron is commonly used to treat iron deficiency anemia in non-dialysis chronic kidney disease (ND-CKD). There is a paucity of information on the potential impact of intravenous iron on patient reported outcome measures, functional status and markers of cardiovascular health. As part of the secondary analysis of this double-blind exploratory randomized controlled trial focusing on patients with iron deficiency (+ /− anemia) and ND-CKD (serum ferritin < 200 µg/L or transferrin saturation ≤ 20% and serum ferritin 200–299 µg/L; CKD stages: 3a-5), 26 patients were randomized in a 1:1 ratio to receive ferric derisomaltose or ferric carboxymaltose. Participants received 1000 mg at baseline and 500–1000 mg at one month to achieve iron repletion. Quality of life and fatigue status were assessed using the Short-Form (36) questionnaire and the fatigue severity scale. Functional status was evaluated using the Duke Activity Status Index and the 1-min-sit-to-stand test. Cardiac markers such as NT-proBNP, Troponin T and pulse wave velocity were monitored. Intravenous iron was associated with similar improvements in most domains of the Short-Form (36) questionnaire, fatigue status, and 1-min-sit-to-stand ability increased significantly by the end of the trial in both groups (*p* < 0.001). Markers of cardiac function remained stable, with no arterial stiffness impact. Longer term studies are required to further evaluate the impact of intravenous iron on quality of life and cardiac safety in patients with ND-CKD.

## Introduction

Anemia (with/without) iron deficiency is a common complication of non-dialysis dependent chronic kidney disease (ND-CKD). This is associated with an impact on mortality, disease progression and hospitalizations^1^. Importantly, patients with anemia of CKD have a greater risk for cardiovascular disease^2^ and frequently report worse health related quality of life outcomes. Indeed as a recent large-scale cross-sectional, prospective cohort study (n = 5,004,957) highlighted, patients with iron deficiency anemia independently correlated with a higher risk of cardiovascular disease^3^. This has been previously noted in two large cohort studies (n = 1,012,014) focusing on cardiovascular associated hospitalizations in CKD, with a relationship between mortality and hospitalization arising in the presence of functional iron deficiency^4,5^. This is not surprising as CKD and uremia represents a state of chronic dysregulation of processes including but not restricted to inflammation and oxidative stress, endothelial function, bone metabolism, autonomic balance and volume control^6^. The degree of injury to these processes can be amplified in the presence of iron deficiency and anemia and may be actually secondary to the extra-hematopoietic functions of iron that include DNA cycle, mitochondrial and non-mitochondrial energy production, neurohormonal abnormalities and decreased muscular oxygen content secondary to reduced myoglobin^7^. In fact, these extra-hematopoietic properties of iron may be related to the symptoms displayed by patients with iron deficiency anemia in CKD.

Iron deficiency with/without anemia in CKD is associated with a significant burden to the patient. It is characterized by debilitating symptoms such as fatigue, dyspnea, angina, headache and loss of concentration. A recent observational study utilizing data for the CKDOPPS registry (n = 2513, ND-CKD) has aptly demonstrated the association between iron deficiency and physical impact. Focusing primarily on the effect of iron deficiency (transferrin saturation < 20% and ferritin > 300 or < 50 ng/mL) on the mental and physical component of KDQOL-36, they concluded that iron deficiency was associated with worse physical component scores. This scoring disadvantage was maintained irrespective of confounders and was most strongly related to transferrin saturation values^8^. Moreover, Finkelstein and colleagues in an analysis utilizing the Short -Form (36) questionnaire (SF-36) in patients with CKD and anemia indicated that at baseline the reported scores in all domains had been lower than the normative value, highlighting the impact of the disease process on quality of life^9^. Iron deficiency has been proposed as key in the symptomatology displayed, as studies suggest that skeletal muscle mitochondrial dysfunction associated with iron depletion may be key in skeletal muscle dysfunction^10,11^.

Intravenous iron is frequently used in the treatment of iron deficiency anemia in patients with ND-CKD. Despite convincing evidence relative to its efficacy and safety based on meta-analyses, a paucity exists on the impact of intravenous iron on patient-reported outcome measures, functional status and cardiovascular markers^12,13^. Additionally concerns remain regarding the implications of iron on cardiovascular toxicity, despite the positive results expressed in studies focusing on patients with heart failure in terms of hospitalization, quality of life and functional status^14–17^. Given the advent of modern intravenous iron compounds such as ferric carboxymaltose (FCM) and ferric derisomaltose (FDI) which share similar efficacy at equivalent dosing but a distinct differential effect on reductions in serum phosphate, research is required on the topic^18–21^.

The “Iron and Phosphaturia – ExplorIRON-CKD” study was primarily designed to explore the differential effect of FDI and FCM on fibroblast growth factor 23 (FGF23) and markers relevant to hypophosphatemia and bone metabolism. Pre-specified secondary outcomes on patient-reported outcome measures, functional status and cardiovascular markers were also collected^22^. We hypothesized that iron repletion would improve functional status and quality of life, while also investigating any differential effect and cardiac consequences.

## Results

### Baseline

A total of 26 patients were randomized into the two groups. Fourteen participants received FDI and 12 received FCM. The baseline group results have been previously discussed^22^. The mean age of the participants was 68 (12.4) years with most of them male. At baseline most patients had absolute iron deficiency (serum ferritin < 100 μg/L and transferrin saturation < 20%) as opposed to functional iron deficiency (61.5% vs. 38.5%). Most participants had advanced CKD with 22 (84.6%) having a CKD stage of 4 or 5 (eGFR < 30 mL/min/1.73 m^2^). All randomized participants received at least one dose of intravenous iron (1000 mg); 10 patients in the FDI group and 11 in the FCM group received a second infusion. At baseline the two groups were comparable with the exception of age, fatigue severity scale (FSS) and heart failure prevalence, whereby the participants in the FDI group were younger (FDI: 63.4 (12.2) vs. FCM: 73.2 (10.8); *p* = 0.040), reported a higher FSS score (FDI: 54.5 (47.8–60.3) vs. FCM: 42.0 (24.5–54.5); *p* = 0.0036) and had a lower prevalence of heart failure (0.026). In addition, a statistically significant difference was noted in terms of QRS interval between the two groups (*p* = 0.018) (Table 1).

**Table 1:** Baseline continuous variable characteristics. Values are presented as mean (SD) or median (IQR).

Variable	Iron group	Value	*p* value	Variable	Iron group	Value	*p* value
Age*/years	Total	67.9 (12.4)		SF36—Physical Function	Total	25.0 (20.0–47.5)	
	FDI	63.4 (12.2)	0.043		FDI	22.5 (17.5–41.3)	0.494
	FCM	73.2 (10.8)			FCM	27.5 (25.0–52.5)	
Body mass index/kg/m^2^	Total	27.8 (25.0–33.4)		SF36—Role limitation (physical)	Total	0.0 (0.0–25.0)	
	FDI	28.8 (26.2–36.3)	0.279		FDI	0.0 (0.0–31.3)	0.297
	FCM	26.8 (23.0–32.7)			FCM	0.0 (0.0–0.0)	
Hemoglobin*/g/L	Total	100.3 (13.5)		SF36—Role limitation (emotional)	Total	33.3 (0.0–100.0)	
	FDI	99.2 (12.2)	0.664		FDI	33.3 (25.0–100.0)	0.231
	FCM	101.6 (15.3)			FCM	0.0 (0.0–91.7)	
Serum Ferritin/μg/L	Total	76.5 (38.7–157.5)		SF36—Energy	Total	25.6 (20.2)	
	FDI	76.5 (25.0–183.5)	0.899		FDI	26.4 (24.8)	0.815
	FCM	72.7 (42.3–146.9)			FCM	24.6 (14.2)	
Transferrin saturation/%	Total	15.0 (11.7–18.5)		SF36—Emotional Wellbeing	Total	68.0 (60.0–81.0)	
	FDI	15.0 (11.0–21.0)	0.781		FDI	66.0 (53.0–84.0)	0.595
	FCM	14.5 (12.0–17.8)			FCM	68.0 (64.0–79.0)	
Creatinine*/μmol/L	Total	269.5 (88.2)		SF36—Social Function	Total	43.8 (25.0–78.1)	
	FDI	277.6 (98.8)	0.626		FDI	43.8 (25.0–90.6)	0.560
	FCM	260.2 (77.3)			FCM	43.8 (28.2–62.5)	
eGFR/mL/min/1.73 m^2^	Total	18.0 (14.0–25.3)		SF36—Pain	Total	45.0 (30.0–67.5)	
	FDI	18.0 (14.0–25.3)	1.000		FDI	45.0 (22.5–70.0)	0.899
	FCM	18.0 (14.0–25.3)			FCM	45.0 (32.5–64.4)	
NT-proBNP/ng/L	Total	875.5 (3033.0)		SF36—General Health	Total	37.5 (23.7–45.0)	
	FDI	856.0 (2555.8)	0.347		FDI	37.5 (17.5–46.3)	0.940
	FCM	1192.5 (3953.5)			FCM	37.5 (30.0–45.0)	
Troponin T/ng/L	Total	36.5 (20.3–60.3)		Duke Activity Status Index/METs	Total	4.9 (4.0–5.7)	
	FDI	28.0 (13.0–66.5)	0.134		FDI	5.1 (4.6–5.7)	0.145
	FCM	41.0 (34.0–55.0)			FCM	4.1 (3.8–5.7)	
Pulse wave velocity (cf) */m/s	Total	8.00 (2.60)		FSS total score	Total	50.0 (35.7–60.0)	
	FDI	8.35 (2.58)	0.516		FDI	54.5 (47.8–60.3)	0.036
	FCM	7.61 (2.73)			FCM	42.0 (24.5–54.5)	
Augmentation Index */%	Total	26.0 (11.8)		FSSVisual Analogue Scale	Total	3.5 (1.8–6.3)	
	FDI	23.4 (11.3)	0.283		FDI	3.0 (1.0–7.0)	0.403
	FCM	28.8 (12.2)			FCM	4.5 (3.0–5.0)	
PR interval/ms	Total	172.0 (156.0–197.5)		1-min-sit-to-stand test */min	Total	16.0 (8.0)	
	FDI	172.0 (153.4–196.5)	0.547		FDI	16.5 (5.9)	0.731
	FCM	173.0 (162.0–212.0)			FCM	15.3 (10.2)	
QRS interval/ms	Total	98.0 (87.0–124.0)					
	FDI	92.0 (80.0–100.0)	0.018				
	FCM	136.0 (88.0–154.0)					
QTc interval/ms	Total	442.0 (426.0–462.0)					
	FDI	440.0 (426.0–451.0)	0.291				
	FCM	460.0 (412.0–496.0)					
Variables noted with *: data presented as mean (SD); otherwise data is presented as median (IQR) p-value represents the statistical significant upon comparison of two groups							

### Patient reported outcome measures

**Figure 1:**
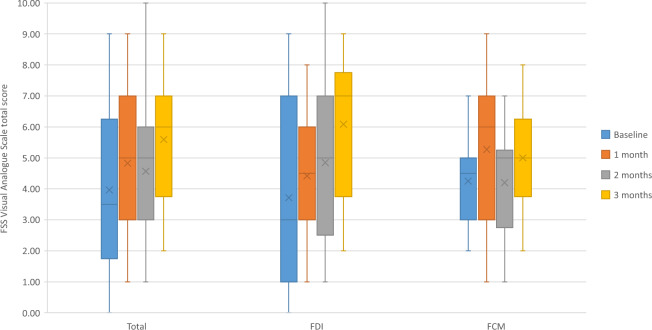
Fatigue Severity Scale Visual Analogue Scale – Box Plot. Legend: The results of the visual analogue scale of the fatigue severity scale are indicated. A significant trend for improvement was noted throughout the population and within the FDI group (*p* = 0.027; *p* = 0.048).

Seven out of eight of the SF-36 domains improved numerically for the total group. Physical function, limitations due to emotional restriction, energy and pain scores demonstrated a significant improvement within the whole cohort (Table 2). No differential effect was noted between the two comparators at each visit, however the significance of the intergroup trends varied. Physical function and energy demonstrated a significant intergroup increase with FDI but not FCM, while for role limitations due to emotional reasons the reverse intergroup trend was observed. No significant difference between the two groups was noted at specific time-points. Fatigue severity scale scores improved in the whole cohort and each group, with a greater but non-significant improvement within the FDI group (supplementary table 1). A significant improvement in fatigue status within the whole cohort and the FDI group was noted based on the FSS Visual Analogue Scale (Baseline: 3.5 (1.8–6.3)/10; 3 months: 6.0 (3.7–7.0)/10; *p* = 0.027 and Baseline: 3.0 (1.0–7.0)/10; 3 months: 7.0 (3.7–8.7)/10; *p* = 0.048 respectively) (Fig. 1).

**Table 2:** SF-36 domain analysis including whole cohort and individualized group.

Visit	Iron group (n)	Mean/Median (SD/IQR)	*p* value	*p* value (within group analysis)	Visit	Iron group (n)	Mean/Median (SD/IQR)	*p* value	*p* value (within group analysis)
**Physical Function**					**Role limitation (physical)**				
Baseline	Total (26)	25.0 (20.0–47.5)			Baseline	Total (26)	0.0 (0.0–25.0)		
	FDI (14)	22.5 (17.5–41.3)	0.494			FDI (14)	0.0 (0.0–31.3)	0.297	
	FCM (12)	27.5 (25.0–52.5)				FCM (12)	0.0 (0.0–0.0)		
1 month	Total (25)	35.0 (20.0–70.0)			1 month	Total (25)	25.0 (0.0–75.0)		
	FDI (14)	35.0 (21.2–67.5)	0.651			FDI (14)	25.0 (6.2–62.5)	0.608	
	FCM (11)	35.0 (15.0–70.0)				FCM (11)	0.0 (0.0–100.0)		
2 months	Total (23)	30.0 (20.0–70.0)			2 months	Total (23)	0.0 (0.0–50.0)		
	FDI (13)	30.0 (25.0–70.0)	0.410			FDI (13)	25.0 (0.0–50.0)	0.832	
	FCM (10)	25.0 (13.7–75.0)				FCM (10)	0.0 (0.0–62.5)		
3 months	Total (22)	37.5 (25.0–80.0)		Total cohort: 0.015	3 months	Total (22)	25.0 (0.0–56.3)		Total cohort: 0.149
	FDI (12)	35.0 (26.3–71.3)	0.582	FDI: 0.042		FDI (12)	37.5 (6.2–50.0)	0.722	FDI: 0.323
	FCM (10)	42.5 (25.0–90.0)		FCM: 0.116		FCM (10)	12.5 (0.0–100.0)		FCM: 0.373
**Role limitation (emotional)**					Energy *				
Baseline	Total (26)	33.3 (0.0–100.0)			Baseline	Total (26)	25.6 (20.2)		
	FDI (14)	33.3 (25.0–100.0)	0.231			FDI (14)	26.4 (24.8)	0.815	
	FCM (12)	0.0 (0.0–91.7)				FCM (12)	24.6 (14.2)		
1 month	Total (25)	66.7 (0.0–100.0)			1 month	Total (25)	40.0 (21.3)		
	FDI (14)	83.4 (41.6–100.0)	0.413			FDI (14)	42.5 (19.2)	0.569	
	FCM (11)	66.7 (0.0–100.0)				FCM (11)	37.3 (24.0)		
2 months	Total (23)	66.7 (33.3–100.0)			2 months	Total (23)	38.9 (21.3)		
	FDI (13)	66.7 (16.6–100.0)	0.784			FDI (13)	38.1 (21.9)	0.836	
	FCM (10)	83.4 (33.3–100.0)				FCM (10)	40.0 (28.8)		
3 months	Total (22)	100.0 (58.4–100.0)		Total cohort: 0.053	3 months	Total (22)	39.5 (18.8)		Total cohort: 0.002
	FDI (12)	100.0 (8.3–100.0)	0.872	FDI: 0.364		FDI (12)	39.2 (19.8)	0.920	FDI: 0.015
	FCM (10)	83.4 (66.7–100.0)		FCM: 0.043		FCM (10)	40.0 (18.6)		FCM: 0.079
**Emotional wellbeing**					**Social function**				
Baseline	Total (26)	68.0 (60.0–81.0)			Baseline	Total (26)	43.8 (25.0–78.1)		
	FDI (14)	66.0 (53.0–84.0)	0.595			FDI (14)	43.8 (25.0–90.6)	0.560	
	FCM (12)	68.0 (64.0–79.0)				FCM (12)	43.8 (28.2–62.5)		
1 month	Total (25)	72.0 (64.0–80.0)			1 month	Total (25)	62.5 (37.5–100.0)		
	FDI (14)	68.0 (61.0–78.0)	0.316			FDI (14)	62.5 (28.1–100.0)	0.976	
	FCM (11)	80.0 (68.0–84.0)				FCM (11)	50.0 (50.0–100.0)		
2 months	Total (23)	72.0 (68.0–84.0)			2 months	Total (23)	50.0 (37.5–87.5)		
	FDI (13)	72.0 (62.0–82.0)	0.208			FDI (13)	50.0 (37.5–93.8)	0.784	
	FCM (10)	80.0 (68.0–89.0)				FCM (10)	62.5 (37.5–90.6)		
3 months	Total (22)	78.0 (56.0–88.0)		Total cohort: 0.780	3 months	Total (22)	75.0 (34.4–100.0)		Total cohort: 0.621
	FDI (12)	78.0 (56.0–87.0)	0.821	FDI: 0.725		FDI (12)	75.0 (37.5–100.0)	0.771	FDI: 0.990
	FCM (10)	74.0 (59.0–88.0)		FCM: 0.764		FCM (10)	68.8 (25.0–90.6)		FCM: 0.248
**Pain**					**General health**				
Baseline	Total (26)	45.0 (30.0–67.5)			Baseline	Total (26)	37.5 (23.7–45.0)		
	FDI (14)	45.0 (22.5–70.0)	0.899			FDI (14)	37.5 (17.5–46.3)	0.940	
	FCM (12)	45.0 (32.5–64.4)				FCM (12)	37.5 (30.0–45.0)		
1 month	Total (25)	55.0 (32.5–87.5)			1 month	Total (25)	35.0 (20.0–50.0)		
	FDI (14)	56.3 (22.5–85.6)	0.786			FDI (14)	30.0 (10.0–48.8)	0.260	
	FCM (11)	45.0 (32.5–100.0)				FCM (11)	35.0 (25.0–60.0)		
2 months	Total (23)	55.0 (32.5–77.5)			2 months	Total (23)	30.0 (20.0–45.0)		
	FDI (13)	55.0 (28.7–90.0)	0.784			FDI (13)	30.0 (17.5–50.0)	0.784	
	FCM (10)	51.3 (30.0–77.5)				FCM (10)	35.0 (20.0–46.3)		
3 months	Total (22)	45.0 (45.0–82.5)		Total cohort: 0.038	3 months	Total (22)	27.5 (18.8–36.3)		Total cohort: 0.245
	FDI (12)	56.3 (35.6–79.4)	0.782	FDI: 0.079		FDI (12)	22.5 (7.5–38.8)	0.346	FDI: 0.439
	FCM (10)	45.0 (45.0–100.0)		FCM: 0.230		FCM (10)	30.0 (23.8–41.3)		FCM: 0.301

* variables characterized by asterisk are described as mean (SD); the remaining variables are described as median (IQR) based on distribution.

### Functional status

**Figure 2:**
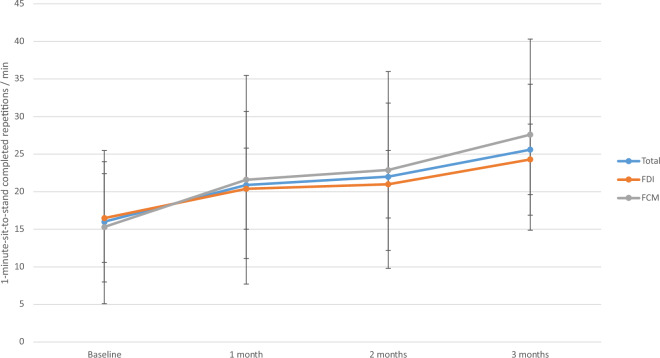
1-min-sit-to-stand test. Legend: Line graph summarizing the results of the 1-min-sit-to-stand test within the study. A significant improvement within the whole cohort (*p* < 0.001) and both groups (*p* < 0.001) was noted. No differential effect was observed.

There was no trend in Duke Activity Status Index (DASI) score in the whole cohort or individual groups (supplementary table 1). No significant differential effect between the two comparators was detected at any point in the trial. The 1-min-sit-to-stand testscores improved significantly and persistently following intravenous iron (*p* < 0.001), irrespective of compound administered indicated by the lack of differential significant effect (Fig. 2).

### Cardiovascular measurements

**Figure 3:**
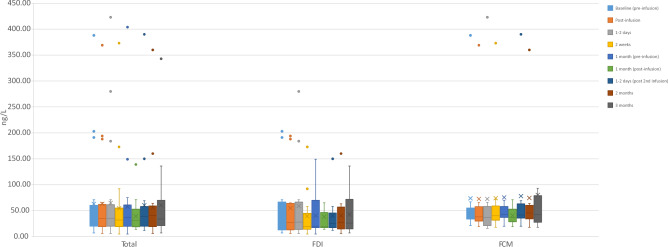
Troponin T concentration – box plot. Legend: The concentrations of troponin T throughout the study in terms of whole cohort and individual groups are presented. No statistically significant trend was witnessed, alongside no differential effect. No indication of cardiac injury was noted.

**Figure 4:**
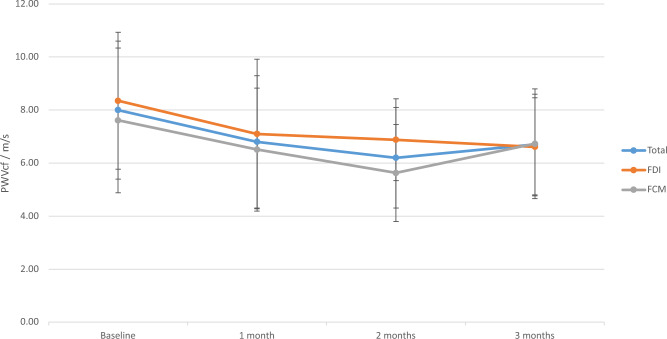
Pulse wave velocity. Legend: Line graph summarizing the results of the pulse wave velocity measurements within the study. A numerical improvement was noted in terms of pulse wave velocity in the whole cohort and individual groups, which did not reach statistical significance. No differential effect was noted.

**Figure 5:**
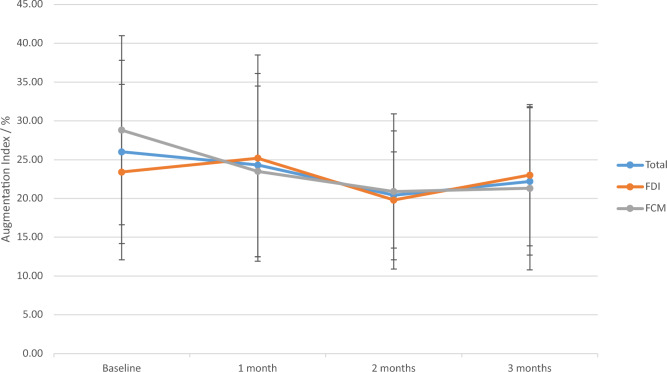
Augmentation index. Legend: Line graph summarizing the results of the augmentation index measurements within the study. No differential effect was noted.

Cardiac markers were elevated at baseline compared to reference ranges, both within the whole population and individual groups. NT-proBNP and Troponin T were unchanged in both groups throughout the study (Table 3) (Fig. 3). There was a small increase in NT-proBNP in the FDI group and a small decrease within the FCM group, however these were both non-statistically significant, numerical changes. Electrocardiograms were not significantly affected in either group by intravenous iron, (supplementary table 2). Pulse wave velocity (PWV) and augmentation index improved numerically but not statistically throughout the study with no differential effect noted (Figs. 4 and 5).

**Table 3:** NT-proBNP concentrations throughout the trial.

Visit	Iron group (n)	Median (IQR)/ng/L	*p* value	*p* value (within group analysis)
Baseline	Total (26)	875.5 (330.0–3363.0)		
	FDI (14)	856.0 (128.5–2684.3)	0.347	
	FCM (12)	1192.5 (415.3–4368.8)		
1 month	Total (23)	1156.0 (298.0–3430.0)		
	FDI (12)	611.0 (189.3–2042.3)	0.091	
	FCM (11)	1420.0 (633.0–5175.0)		
2 months	Total (22)	1386.0 (335.3–4965.3)		
	FDI (12)	1204.0 (239.3–2581.3)	0.254	
	FCM (10)	2548.0 (398.3–8595.3)		
3 months	Total (20)	967.0 (299.5–3275.5)		Total cohort: 0.626
	FDI (11)	1041.0 (262.0–2224.0)	0.710	FDI: 0.921
	FCM (9)	893.0 (370.0–4977.0)		FCM: 0.449

## Discussion

The “Iron and Phosphaturia – ExplorIRON-CKD” study has demonstrated a trend for improvement in quality of life and functional status following administration of intravenous iron. A significant improvement in the whole cohort was seen in terms of physical function, energy, emotional role limitation, and pain using the results of the SF-36, alongside a significant improvement in functional status and fatigue as delineated by 1-min-sit-to-stand test and FSS visual analogue scale respectively. Intravenous iron did not lead to any detrimental effect on surrogate markers of cardiac strain and cardiomyocite damage, whilst some beneficial result was noted on vascular function/arterial stiffness.

As suggested by Strauss and Auerbach, fatigue resolution is best monitored through a combination that allows the investigation of a continuum between vitality and fatigue^23^. The combination of SF-36 and FSS has allowed us to assess this continuum through a fatigue-specific questionnaire alongside a multifaceted assessment of various domains that are associated with elements of fatigue. At baseline, the SF-36 scores noted were below the 50% cut-off value, indicating the impact that CKD has on patients. In addition, it was below the previously reported values by Finkelstein and colleagues, potentially highlighting the implications of iron deficiency anemia in this patient group^9^. This was also the case in FSS^24^. Most domains relevant to physical function improved significantly in both the whole-cohort and the FDI subgroup, suggesting that iron repletion (but not necessarily anemia resolution) can be associated with improved physical function. This was also the case for the energy (vitality) domain of the SF-36. The absence of significance within the FCM group may be explained by the recent findings commented in PHOSPHARE-IBD, where a slower, lesser improvement in FACIT-fatigue was seen where FCM was administered compared to FDI despite similar changes in hemoglobin with equivalent doses^18^. Analysis of this study suggests that this, with analysis suggesting that this could be phosphate dependent, where fatigue and muscle weakness are common symptoms of hyposphatemia^18^. These trends were replicated in the FSS visual analogue scale, with a significant improvement within the whole cohort and the FDI group. Nonetheless, no significant differential effect was witnessed—this may be partially due to the extent of kidney dysfunction in the randomized population limiting the potential for hyperphosphaturic hypophosphatemia. Indeed no hypophosphatemia as per protocol was detected (serum phosphate concentration < 0.65 mmol/L). However, it is important to aknowledge that the participants receiving FCM were significantly older than those receiving FDI with comorbidities such as heart failure which may have impacted their response to intravenous iron supplementation.

Functional status improved as indicated through the 1-min-sit-to-stand test scores. Similar to quality of life, the baseline scores achieved were lower than the ones previously reported in literature relevant to CKD, potentially noting the effect of iron deficiency anemia^25^. A statistically significant improvement throughout the trial was seen in all groups, with no differential effect potentially underlining the effect of iron repletion irrespective of compound used. It is also important to note that the scores achieved in both groups and the whole cohort were above the minimal detectable change previously described by Wilkinson and colleagues^25^. These results appear to complement the previously completed “Iron and the Heart” multicenter randomized controlled trial which noted an increase in the 6-min-walking test distance in participants with ND-CKD and iron deficiency that received FDI^26^. The trend observed in the aforesaid study was not significant; this could be secondary to the small sample size and the high baseline function of the randomized participants. The DASI did not exhibit any significant trends during the study, but a beneficial signal was seen with FCM. This may be due to the group dynamics. The FDI group was composed by three participants requiring hemodialysis during the study, a procedure associated with worse DASI scores than pre-dialysis^27^.

Cardiovascular markers including NT-proBNP and Troponin T remained stable throughout the trial and was similar in both groups. Previous studies related to ND-CKD and iron deficiency have noted a non-significant decrease in NT-proBNP following intravenous iron administration (1000 mg FDI) (baseline: 422.5 (881.9) pg/mL to 1-month: 242.5 (209.1) pg/mL)^26^. Iron sucrose has been previously associated with a significant decrease of NT-proBNP in patients with ND-CKD and heart failure^28^. In the present study, this appeared to be the case in patients that received FCM but not FDI. This, nonetheless, may be related to the sample of the patients within FDI (greater number of participants in the FDI group requiring dialysis) and not signify a differential effect. Outside interference such as increased diuretic therapy in certain patients cannot be excluded. Troponin T was not affected by the administration of intravenous iron and that potentially suggests a degree of cardiac safety in the use of high dose iron administration. In the absence of comparative/observational in-vivo studies, animal models of kidney injury and iron deficiency have demonstrated improved cardiac mitochondrial function with attenuation of oxidative/nitrosative stress secondary to a pre-conditioned/primed oxidant status existing in iron deficiency and kidney disease^11,29,30^. Indeed, previous results relevant to FDI have suggested that the use of high dose intravenous iron (1000 mg) is not associated with increased oxidative stress or inflammation, that are pathological processes directly implicated in cardiac toxicity^31,32^. Any analysis of electrocardiographical parameters and extrapolation of the results is limited by the presence of implantable cardiac devices and arrhythmias in a number of the participants. Similar to the HOMeaFers randomized controlled study, comparing FDI and FCM in individuals with iron deficiency no differential effect was witnessed between the two intravenous iron preparations^33^.

The results of the present trial are also in agreement with previously published evidence pertaining to vascular function following administration of intravenous iron in patients with ND-CKD. Such trends highlight a potential mechanistic safety despite concerns regarding oxidative stress and endothelial damage. A previous pooled analysis has noted no worsening in PWV(cf) or Augmentation Index following administration of 1000 mg FDI in ND-CKD^34^. Similarly, intravenous iron sucrose (300 mg) administered in patients on peritoneal dialysis was not associated with increased vascular reactivity^35^.

This trial had several limitations including a small number of participants with no ethnic minority groups and therefore generalizability of the results is not possible. In addition, the possibility of errors upon completion of the questionnaires provided cannot be excluded. The small numbers may represent the reason behind the lack of any differential effect noted. In addition, certain differences between the two groups at baseline may account for the lack of significant findings of the DASI scores or the differential effect in terms of FSS scores. Indeed participants in the FCM arm were significantly older with prolonged QRS complexes and greater incidence of heart failure. It is also possible that detection of significance within the FCM group was hindered by its small size. Moreover, the trial took place throughout the COVID-19 pandemic which was characterized by higher psychological stress in patients with CKD^36,37^. Given that secondary outcomes focused on patient reported outcomes, the absence of placebo arm may be also considered as a limitation; placebo response has previously been documented as an important phenomenon in patient reported outcome measures and this is something not addressed in the present study^38^.

Nevertheless, the “Iron and Phosphaturia – ExplorIRON-CKD” was a double-blind randomized controlled trial, providing valuable results for direct future research. The results are suggestive of improvement in functional status and quality of life following intravenous iron infusion. To our knowledge, this is the first trial to have provided evidence supporting the notion of intravenous iron administration leading to 1-min-sit-to-stand test improvement in patients with ND-CKD and iron deficiency anemia. In addition, this exploratory study is the first to compare to two modern intravenous iron in patients with advanced kidney dysfunction (median eGFR 18 mL/min/1.73 m^2^), displaying results that complement the efficacy of supplementation beyond hematological markers, into aspects of quality of life. Moreover, the results of cardiovascular variables support the notion of mechanistic safety and potential improvement of certain processes (such as arterial stiffness) following alleviation of iron deficiency. Additionally, it was created based on research priorities and targets delineated both by patient groups and large research groups^39,40^.

As part of the secondary outcome analysis of the current trial, no significant differential effect was noted in quality of life measures, functional status and cardiovascular biomarkers. An improvement was witnessed in most domains of the Short-Form (36) questionnaire, fatigue status, and 1-min-sit-to-stand ability increased significantly by the end of the trial in whole population. These results provide further evidence on the neutrality of modern intravenous iron compounds in terms of cardio and myotoxicity and potential benefit in terms of functional status. This is important clinically, as CKD and iron deficiency anemia are considered risk factors to cardiovascular disease, with prior concerns rending iron as a pro-oxidant substance. These concerns may explain previous publication results suggesting persistent under-dosing in patients with CKD requiring intravenous iron, therefore potentially limiting the benefit that could be obtained by accurate supplementation^41^. Some particular signals were noted (larger improvement in aspects relevant to fatigue, physical function) with FDI that may be related to the cascade effects of FCM on the metabolism of FGF23. Further studies are required to elicit whether any differential effect exists secondary to intravenous iron compound used in this patient group, as has been demonstrated in patients with inflammatory bowel disease. Moreover, further research could help identify, via stratification, whether a differential response in terms of functional status and quality of life exists dependent on background kidney function, anemia status, and mode of iron supplementation. The eagerly awaited “Iron and Muscle” trial could provide further answers as to the potential improvement in exercise capacity of patients with iron deficiency and ND-CKD, a signal alluded in the present trial^42^.

## Methods

The “Iron and Phosphaturia – ExplorIRON-CKD” (EudraCT number: 2019-004370-26) took place according to the Declaration of Helsinki and the Good Clinical Practice guidelines. Clinical trial authorization was provided by the Health Research Authority and the Medicines and Healthcare Products Regulatory Agency of the United Kingdom. The trial received the favorable opinion of the Research Ethics Committee Leeds West (20/YH/0005). All participants were enrolled only after full informed consent was taken.

The baseline results and methodology of the present study have been previously published^22^. “Iron and Phosphaturia – ExplorIRON-CKD” was a double blind randomized exploratory study taking place in a tertiary university hospital in the United Kingdom, primarily investigating the potential differential effect of two modern intravenous iron preparations on FGF23 and markers of bone metabolism. As a secondary outcome, the study explored the impact of high dose intravenous iron supplementation on the quality of life, functional status and markers of cardiovascular status of the participants. Patients with iron deficiency with/without anemia (serum ferritin < 200 μg/L and/or transferrin saturation < 20% and serum ferritin 200–299 μg/L) and stable ND-CKD (3a-5; estimated glomerular filtration rate (eGFR) < 60 mL/min/1.73 m^2^) were randomized in a 1:1 ratio to receive 1000 mg of FDI or FCM. A repeat infusion of either 500/1000 mg (dependent on baseline hematinic parameters and weight) was administered a month later.

### Patient reported outcome measures

Patient reported outcome measures were monitored at baseline, 1 month following first infusion, 2 months following first infusion and 3 months following first infusion. The SF-36 version 1.0 questionnaire and the FSS were used to assess quality of life. The SF-36 is a 36 question based survey that assesses eight variable including vitality, physical functioning, bodily pain, physical role functioning, social functioning, emotional functioning, mental health and general health^43^. Higher SF-36 questionnaire scores indicate better quality of life. The FSS is a nine-item questionnaire that is scored on a seven-point Likert scale, specifically assessing elements pertaining to fatigue. These include motivation, impact of exercise, fatigability, interference with life, impact on normal function, fatigue frequency, impact on activities and fatigue interference with social life^44^. The FSS can be scored as a total and on a visual analogue scale. The greater the FSS, the greater the fatigue experienced by the patient, whilst a lower visual analogue scale score is associated with worse fatigue.

### Functional status

This was monitored through the DASI and the 1-min-sit-to-stand test. Measurements took place on baseline, 1 month, 2 months and 3 months following initial infusion. The DASI is a 12-item questionnaire developed to non-invasively evaluate the functional capacity of participants (VO_2_ peak), a surrogate measure of aerobic fitness. The scores were converted and are reported as metabolic cost of task (MET). The DASI score is considered to have high reliability in CKD and advocated by the Renal Association UK for the estimation of functional capacity^45^. The 1-min-sit-to-stand test was utilized as a measure of lower extremity, exercise capacity and fatigability^46^. Participants were asked to sit and stand up as many times as possible within one minute from a chair of a standard height (46 cm with no armrests) with arms crossed across chest. Only full motion (complete sitting) was counted and recorded. All participants were informed that they could stop as necessary. The 1-min-sit-to-stand test has been previously demonstrated as valid and reliable in CKD^27^.

### Cardiovascular measurements

N-terminal pro-hormone BNP (NT-proBNP) and Troponin T were measured, in addition to electrocardiography. Cardiac strain was non-invasively monitored at baseline, 1 month, 2 months and 3 months following initial administration through NT-proBNP. The Roche cobas e411 analyzer was used alongside the Elecsys proBNP II immunoassay (Roche Diagnostics, Risch-Rotkreuz, Switzerland). Troponin T, a widely accepted marker of ischemia and myocyte damage, was measured at baseline, prior to first infusion and 30 min following first infusion, 1–2 days following first infusion, 2 weeks following first infusion, 1 month (before and after second administration), 1–2 days following second infusion, 2 months and 3 months following initial infusion. The Elecsys Troponin T assay was used (Roche Diagnostics, Risch-Rotkreuz, Switzerland. Electrocardiography was performed at baseline, 1–2 days following first infusion, 1 month, 2 months and 3 months after initial administration to identify any arrhythmias, with measurements of PR, QRS and QTc intervals. Aortic stiffness assessed using measurement of pulse wave velocity carotid-femoral (PWVcf) and Augmentation Index using the Enverdis^®^ Vascular Explorer (Enverdis GmbH Medical Solutions, Jena, Germany). Measurements took place at baseline, 1 month, 2 months and 3 months following initial intravenous iron infusion. The American Heart Association advocates the use of PWVcf as a surrogate marker of arterial stiffness^47^.

### Statistical analysis

All randomized participants were included in the statistical analysis based on an intention-to-treat approach. Whole cohort and subgroup analysis took place to identify any signals of differential effects. Categorical data is summarized as mean (standard deviation (SD)) or median (interquartile range (IQR)), whilst categorical data is presented as number (%). The Shapiro–Wilk test was used to assess normality of distribution. The independent T-test and Mann–Whitney U tests were used for between group analyses (i.e. FCM vs FDI). The Skillings-Mack test was performed to identify any longitudinal trends within groups. Given the small sample, adjustments for multiple comparisons were not performed. A statistical software package was used for the analysis of data (IBM SPSS Statistics Version 26, IBM Corp. 2019). Statistical significance was deduced at p-value < 0.05.

### Supplementary Information

Supplementary Tables.

## Data Availability

The data associated with the paper are not publicly available but are available from the corresponding author on reasonable request with the relevant permissions and agreement of the Research and Development Department of the Hull University Teaching Hospitals NHS Trust that served as the sponsor for the trial. Further enquiries can be directed to the corresponding author.
