# Supplementary material for: Patient reported outcome measures and cardiovascular outcomes following high dose modern intravenous iron in non-dialysis dependent chronic kidney disease: secondary analysis of ExplorIRON-CKD
Source: Sci Rep. 2023 Oct 26;13:18401. doi: 10.1038/s41598-023-44578-6 (PMC10603042; doi:10.1038/s41598-023-44578-6)
Supplement: Supplementary file 1 — Supplementary Tables. [file 41598_2023_44578_MOESM1_ESM.docx]

| **Supplementary table 1: FSS Total score and DASI scores throughout the study** | | | | | | | | | |
| --- | --- | --- | --- | --- | --- | --- | --- | --- | --- |
| **Visit** | **Iron group (n)** | **Median (IQR)** | **p-value** | **p- value (within group analysis)** | **Visit** | **Iron group (n)** | **Median (IQR)** | **p-value** | **p- value (within group analysis)** |
| **FSS total score** | | | | | **DASI / METs** | | | | |
| Baseline | Total (26) | 50.0 (35.7-60.0) |  |  | Baseline | Total (26) | 4.9 (4.0-5.7) |  |  |
|  | FDI (14) | 54.5 (47.8-60.3) | 0.036 |  |  | FDI (14) | 5.1 (4.6-5.7) | 0.145 |  |
|  | FCM (12) | 42.0 (24.5-54.5) |  |  |  | FCM (12) | 4.1 (3.8-5.7) |  |  |
| 1 month | Total (23) | 48.0 (37.0-58.0) |  |  | 1 month | Total (23) | 4.6 (4.3-5.7) |  |  |
|  | FDI (12) | 52.5 (34.8-60.3) | 0.424 |  |  | FDI (12) | 4.8 (4.4-7.4) | 0.211 |  |
|  | FCM (11) | 44.0 (37.0-56.0) |  |  |  | FCM (11) | 4.4 (4.1-5.6) |  |  |
| 2 months | Total (23) | 49.0 (40.0-58.0) |  |  | 2 months | Total (22) | 5.1 (4.4-6.5) |  |  |
|  | FDI (13) | 55.0 (41.0-60.0) | 0.284 |  |  | FDI (12) | 5.1 (4.7-7.4) | 0.228 |  |
|  | FCM (10) | 48.0 (35.5-53.5) |  |  |  | FCM (10) | 4.7 (4.0-6.3) |  |  |
| 3 months | Total (22) | 47.5 (35.5-57.3) |  | Total cohort : 0.884 | 3 months | Total (22) | 5.1 (4.7-6.7) |  | Total cohort: 0.294 |
|  | FDI (12) | 49.5 (32.8-57.8) | 0.771 | FDI: 0.653 |  | FDI (12) | 5.1 (4.7-7.1) | 0.346 | FDI: 0.376 |
|  | FCM (10) | 42.5 (35.5-55.5) |  | FCM: 0.249 |  | FCM (10) | 4.8 (4.5-5.9) |  | FCM: 0.419 |

| **Supplementary table 2: Electrocardiography results** | | | | | | | | | |
| --- | --- | --- | --- | --- | --- | --- | --- | --- | --- |
| **Visit** | **Iron group (n)** | **Mean/Median (SD/IQR)** | **p-value** | **p- value (within group analysis)** | **Visit** | **Iron group (n)** | **Mean/Median (SD/IQR)** | **p-value** | **p- value (within group analysis)** |
| **PR interval / ms** | | | | | **QTc / ms** | | | | |
| Baseline | Total (20) | 172.0 (156.0-197.5) |  |  | Baseline | Total (25) | 442.0 (426.0-462.0) |  |  |
|  | FDI (14) | 172.0 (153.4-196.5) | 0.547 |  |  | FDI (14) | 440.0 (426.0-451.0) | 0.291 |  |
|  | FCM (6) | 173.0 (162.0-212.0) |  |  |  | FCM (11) | 460.0 (412.0-496.0) |  |  |
| 1-2 days | Total (15) | 166.0 (144.0-192.0) |  |  | 1-2 days | Total (21) | 442.0 (413.0-483.0) |  |  |
|  | FDI (11) | 168.0 (144.0-192.0) | 0.571 |  |  | FDI (12) | 439.5 (416.0-449.0) | 0.219 |  |
|  | FCM (4) | 160.0 (130.0-187.0) |  |  |  | FCM (9) | 483.0 (417.0-501.0) |  |  |
| 1 month | Total (15) | 172.0 (160.0-186.0) |  |  | 1 month | Total (20) | 430.0 (418.5-471.5) |  |  |
|  | FDI (10) | 173.0 (160.0-191.0) | 0.679 |  |  | FDI (10) | 429.5 (421.0-436.0) | 0.579 |  |
|  | FCM (5) | 164.0 (153.0-191.0) |  |  |  | FCM (10) | 455.0 (407.0-484.0) |  |  |
| 2 months | Total (14) | 171.0 (164.5-190.5) |  |  | 2 months | Total (19) | 426.0 (418.0-465.0) |  |  |
|  | FDI (9) | 176.0 (167.0-191.0) | 0.606 |  |  | FDI (10) | 423.0 (417.0-432.0) | 0.156 |  |
|  | FCM (5) | 170.0 (153.0-191.0) |  |  |  | FCM (9) | 465.0 (409.0-495.0) |  |  |
| 3 months | Total (13) | 168.0 (155.0-183.0) |  | Total cohort: 0.026 | 3 months | Total (17) | 438.0 (414.0-455.0) |  | Total cohort: 0.861 |
|  | FDI (8) | 171.0 (164.0-189.0) | 0.354 | FDI: 0.116 |  | FDI (8) | 428.0 (415.0-451.0) | 0.606 | FDI: 0.458 |
|  | FCM (5) | 158.0 (148.0-178.0) |  | FCM: 0.079 |  | FCM (9) | 444.0 (412.0-499.0) |  | FCM: 0.113 |
| **QRS / ms** | | | | |  | | | | |
| Baseline | Total (25) | 98.0 (87.0-124.0) |  |  |  |  |  |  |  |
|  | FDI (14) | 92.0 (80.0-100.0) | 0.0.18 |  |  |  |  |  |  |
|  | FCM (11) | 136.0 (88.0-154.0) |  |  |  |  |  |  |  |
| 1-2 days | Total (21) | 94.0 (84.0-123.0) |  |  |  |  |  |  |  |
|  | FDI (12) | 92.0 (82.5-101.5) | 0.041 |  |  |  |  |  |  |
|  | FCM (9) | 128.0 (91.0-162.0) |  |  |  |  |  |  |  |
| 1 month | Total (20) | 97.0 (88.5-128.5) |  |  |  |  |  |  |  |
|  | FDI (10) | 93.0 (83.0-103.0) | 0.063 |  |  |  |  |  |  |
|  | FCM (10) | 115.0 (92.0-162.0) |  |  |  |  |  |  |  |
| 2 months | Total (19) | 98.0 (86.0-132.0) |  |  |  |  |  |  |  |
|  | FDI (10) | 90.0 (186.0-101.0) | 0.156 |  |  |  |  |  |  |
|  | FCM (9) | 138.0 (84.0-157.0) |  |  |  |  |  |  |  |
| 3 months | Total (17) | 96.0 (89.0-142.0) |  | Total cohort: 0.422 |  |  |  |  |  |
|  | FDI (8) | 90.0 (85.0-100.0) | 0.059 | FDI: 0.351 |  |  |  |  |  |
|  | FCM (9) | 130.0 (92.0-181.0) |  | FCM: 0.400 |  |  |  |  |  |
